# Preoperative Neutrophil-to-Lymphocyte Ratio Is a Predictor of Recurrence following Thermal Ablation for Recurrent Hepatocellular Carcinoma: A Retrospective Analysis

**DOI:** 10.1371/journal.pone.0110546

**Published:** 2014-10-20

**Authors:** Xin Li, Zhiyu Han, Zhigang Cheng, Jie Yu, Shirong Liu, Xiaoling Yu, Ping Liang

**Affiliations:** Department of Interventional Ultrasound, Chinese PLA General Hospital, Beijing, China; West German Cancer Center, Germany

## Abstract

**Purpose:**

The aim of this study is to determine the predictive value of preoperative blood neutrophil-to-lymphocyte ratio (NLR) for recurrence in recurrent hepatocellular carcinoma (RHCC) patients following thermal ablation.

**Material and Methods:**

This retrospective study enrolled 506 RHCC patients who underwent thermal ablation from April 2006 to April 2014. The clinicopathological parameters including NLR were evaluated to identify predictors of recurrence rate after thermal ablation. A Cox multiple regression analysis was performed to determine the parameters that predicted recurrence in RHCC patients. The best cutoff value of NLR was determined with time-dependent receiver operating characteristic (ROC) curve analysis. The recurrence-free survival (RFS) rate was determined in patients with high and low NLR.

**Results:**

The multivariate Cox proportional hazard model analysis showed that NLR was a prognostic factor in recurrence-free survival. NLR ≥2.14 was evaluated (AUROC = 0.824; *P*<0.001), and 183 of 506 patients (36.2%) had a NLR of more than 2.14. During the follow-up period (12–96 months), the 1-, and 3- year recurrence rates were 20.7% and 31.6% in low NLR group, respectively. These recurrence rates were significantly less than those in the high NLR group (57.9% and 82.5%, respectively) (*P*<0.001). A recurrence-free survival analyses demonstrated that the RFS in the low NLR group (67.2%) was significantly higher than that in the high NLR group (13.1%) (*P*<0.001).

**Conclusion:**

Our results show that preoperative NLR is a predictor for re-recurrence after thermal ablation in RHCC patients. Additionally, patients with NLR <2.14 have a lower recurrence rate, which may improve the clinical management of RHCC patients.

## Introduction

Hepatocellular carcinoma (HCC) is a malignant disease with a poor prognosis worldwide. HCC occurs as a result of chronic inflammation and cirrhosis [1]–[2]. However, a major problem following treatment is tumor recurrence caused by intrinsic HCC biological features [3]. The immune status and inflammation processes of HCC influence the survival and recurrence after treatment [4]–[6]. When HCC recurs, hepatic function and immunity decline. Many recurrent HCC (RHCC) patients are not eligible for surgery; thus, thermal ablation therapy represents the treatment of choice and provides excellent clinical results. Additionally, thermal ablation therapy is less invasive than surgical resection [7]–[9].

Recent evidence suggests that the presence of systemic inflammation and immune status correlates with poorer cancer-specific survival in certain cancers. The neutrophil/lymphocyte ratio (NLR) is a simple index available in the clinic. A high NLR predicts poor survival in patients with various cancers including colorectal cancer, HCC, intrahepatic cholangiocarcinoma, and pancreatic cancer [5]–[6], [10]–[13]. However, this factor has not been extensively examined in patients with RHCC following thermal ablation. Determining the risk of recurrence in RHCC patients after thermal ablation represents a central issue in improving clinical management.

The aims of this retrospective study were to determine the best cutoff value for preoperative NLR in RHCC patients following thermal ablation and to evaluate whether the new cutoff value for NLR correlates with tumor recurrence. Furthermore, we established a simple preoperative prognostic scoring system that may aid in the selection of patients who would benefit most from thermal ablation for RHCC.

## Materials and Methods

### Patient population

Between April 2005 and April 2014, 506 RHCC patients (420 men and 86 female, age ranged 28–85 year, mean± standard deviation = 59.2±11.1 year) with 1032 HCC nodules detected by either conventional sonography or contrast enhanced US/CT/MRI were enrolled in this study. All patients received thermal ablation treatment with curative intention in our institution. The protocol was proposed in accordance with the patients’ medical history, CT/MRI imaging and laboratory results. The maximum diameter of lesions in our study ranged from 1.0–7.9 cm (2.6±1.4 cm). The patient’s prior treatments included liver resection (n = 93), transarterial chemoembolization (TACE) (n = 175) and ablation (radiofrequency, microwave and ethanol) (n = 238). The types of virus infections included the following: HBV infected 331 patients, HCV infected 49 patients, HBV+HBV infected 9 patients, and no infection 67 patients. The Child-Pugh score for RHCC patients was evaluated as A (n = 454) and B (n = 52). The NLR was defined as the absolute neutrophil count divided by the absolute lymphocyte count. The final diagnosis of HCC was determined by pathological examination of biopsy specimens. This study was approved by our institutional human research review committee (Medical Ethics Committee of Chinese PLA General Hospital). Written informed consent was obtained from all patients.

### Pre-ablation examination

The inclusion criteria for our study were the following: 1) non-resectable tumors or patient refusal to undergo surgery; 2) single HCC lesion ≤8 cm; 3) more than two HCC lesions with a maximum diameter ≤4 cm; 4) absence of portal vein thrombosis or extrahepatic metastases; 5) prothrombin time <25 s; 6) prothrombin activity >40%; 7) platelet count >40 cells×10^9^/L. The exclusion criteria were the following: 1) severe cardiopulmonary disease; 2) serious renal function failure; 3); severe liver function failure such as large-volume ascites and hepatic encephalopathy; 4) active severe infection. All patients received conventional ultrasound, CEUS (Contrast-enhanced ultrasound), contrast-enhanced CT and/or MRI to delineate the target tumor before ablation.

### Microwave ablation protocol

All treatments were performed in our institution. Prior to ablation, a US/CEUS-guided biopsy was performed using an automatic biopsy gun with an 18-gauge cutting needle under local anesthesia with 1% lidocaine. During each biopsy, 2 to 3 punctures were performed. Subsequently, the antennas were percutaneously inserted into the tumor and placed in the desired location by guidance with US or CEUS. A single antenna was used for lesions <1.7 cm in diameter. However, for tumors ≥1.7 cm in diameter, two or more antennas were used. General anesthesia (propofol, 6–12 mg/kg per hour; ketamine, 1–2 mg/kg) was employed after antenna placement and ablation were performed. If the lesion was adjacent to the bile duct, gallbladder or bowel (≤5 mm), a 21-gauge thermocouple was placed percutaneously at a designated location to monitor temperature in real time [14]. The temperature was kept at 50–54C for no longer than 3 min using intermittent microwave emissions [15]. If the lesion was located near the diaphragmatic dome, then artificial ascites was used. To completely ablate the tumor lesions larger than 5 cm or very close to the bile duct, gallbladder or bowel, ethanol was injected into the marginal tumor tissue through a 21G PTC needle during the ablation. When the hyper-echo overlapped the whole lesion, the antennas were withdrawn. The needle tracks were routinely cauterized to avoid bleeding and tumor seeding.

### Follow-up

A complete ablation was defined as the absence of enhancement in any areas of the lesion on enhanced images obtained at 1-month after thermal ablation. The follow-up period continued until April 2014 and ranged from 12–96 months. Routine contrast-enhanced US and CT and/or MRI were repeated during the follow-up period to monitor recurrence or metastasis beginning 3 months after ablation. Monitoring then occurred at 6-month intervals. The follow-up was terminated when either the contrast-enhanced imaging or US-guided core needle biopsy was positive.

### Statistical analysis

The data were analyzed using SPSS for Windows (Version 16.0). All data are expressed as the mean ± standard deviation. The preoperative clinical parameters that affected recurrence-free survival were entered into the multivariate Cox proportional hazard model to determine their independent effect. The best cutoff value of preoperative NLR was determined by time-dependent receiver operating characteristic (ROC) curve analysis [16]. Independent χ2 tests were used to compare categorical variables. Continuous variables were compared using unpaired T-tests. The recurrence-free survival (RFS) curves were analyzed using the Kaplan-Meier method and compared using the log-rank test. All *P*- values of less than 0.05 were considered to be statistically significant.

## Results

### Correlation between NLR and RHCC recurrence following thermal ablation

To determine whether NLR was correlated with HCC recurrence after thermal ablation in RHCC patients, we performed multivariate Cox proportional hazard model analysis. The statistically significant predicative factors for recurrence identified by multivariate analyses are shown in Table 1. Among the 12 clinical parameters analyzed (pre-treatment, age, gender, tumor number, tumor size, type of hepatitis, cirrhosis, differentiation, AFP, CHE, NLR and Child-Pugh score), larger tumor number, high AFP, low CHE and high NLR were independent predicative factors for recurrence in RHCC patients.

**Table 1:** Multivariate Cox regression analysis of factors related to recurrence-free survival of RHCC patients.

Clinical parameter	B	SE	Wals	df	Sig.	Exp (B)	95% C.I for Exp(B)	
							Lower	Upper
Pre-treatment	.088	.084	1.092	1	.296	1.091	.926	1.286
Age (year)	.003	.006	.299	1	.584	1.003	.992	1.015
Gender	−.095	.162	.346	1	.556	.909	.662	1.249
Tumor number	.149	.052	8.292	1	.004*	1.161	1.049	1.285
Size of tumor (cm)	−.027	.047	.331	1	.565	.974	.889	1.067
Type of hepatitis	.073	.069	1.139	1	.286	1.076	.940	1.232
Cirrhosis	.244	.196	1.548	1	.213	1.277	.869	1.875
Differentiation	−.108	.098	1.222	1	.269	.898	741	1.087.
AFP(µg/L)	.000	.000	13.445	1	.000*	1.000	1.000	1.000
CHE (u/L)	.000	.000	5.727	1	.017*	1.000	1.000	1.000.
NLR	.110	.013	75.042	1	.000*	1.117	1.089	1.145
Child-Plug	−.102	.227	.204	1	.651	.903	.579	1.407

Note: *P<0.05.

Abbreviations: SE, standard error; CI, confidence interval; AFP, α-fetoprotein; CHE: serum cholinesterase; NLR, neutrophil-to-lymphocyte ratio; df, degrees of freedom.

### Selection of the best cutoff value for NLR in RHCC patients

**Figure 1:**
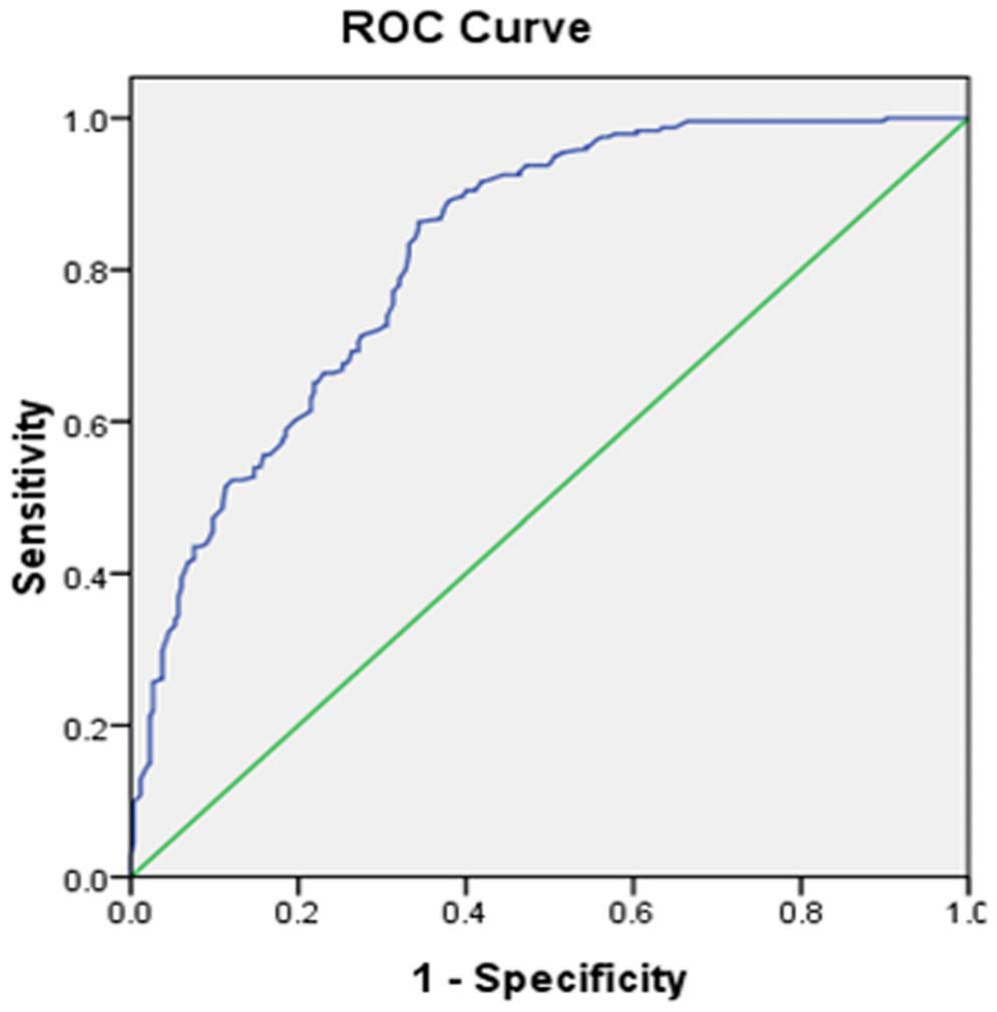
Receiver operating curves analysis for NLR predicating recurrence in RHCC patients following thermal ablation. NLR ≥2.14 was considered to be evaluated (AUROC = 0.824; *P*<0.001).

To analyze the predicative NLR value for tumor re-recurrence in RHCC patients following thermal ablation, we performed a time-dependent receiver operating characteristic (ROC) curve. An NLR of 2.14 was the best cutoff value to predict recurrence after thermal ablation in RHCC patients (AUROC was 0.82, *P*<0.001) (Figure 1). Therefore, we utilized an NLR cutoff of 2.14 as a risk factor for RHCC recurrence. All patients were divided into either a low (<2.14) NLR group (n = 323) or a high (≥2.14) NLR group (n = 183).

### Comparisons of recurrence rate in low and high NLR groups of RHCC patients

During the follow-up period, the 1- and 3-year recurrence rates were 20.7% and 31.6% in low NLR group, respectively. This result was significantly less than that in the high NLR group (57.9% and 82.5%) (*P*<0.001). Therefore, NLR can predict the risk of re-recurrence in RHCC patients following thermal ablation.

### Comparisons of the recurrence-free survival (RFS) between low and high NLR groups of RHCC patients

**Figure 2:**
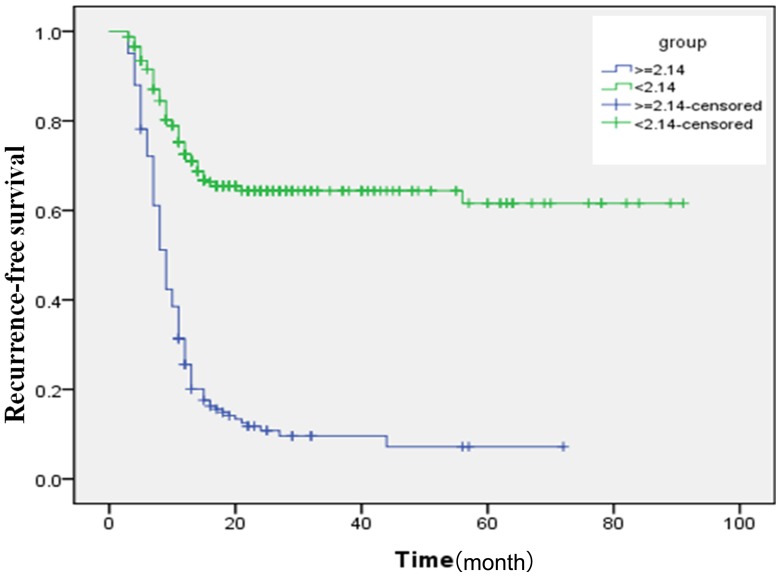
Comparison of recurrence-free survival rates in the low (<2.14) and high (≥2.14) NLR groups. The recurrence-free survival rate was significantly higher in the low NLR group than in the high NLR group (*P*<0.001).

To compare the difference of RFS rate between the low and high NLR groups of RHCC patients, we eliminated the differences of basic clinical parameters between the two groups. The 11 clinical parameters of the low NLR and high NLR groups are compared in Table 2 and 3. No significantly different factors were found between the two groups. The RFS rate after thermal ablation was 67.2% in the low NLR group and 13.1% in the high NLR group (*P*<0.001). The recurrence-free survival rates of patients in the low and high NLR groups are shown in Figure 2. The RFS rate was significantly higher in the low NLR group than in the high NLR group (*P*<0.001).

**Table 2:** Comparison of clinical parameters of RHCC patients between the low and high NLR groups (chi-square test).

Clinical parameter	No	NLR≥2.14 (N = 183)	NLR<2.14 (N = 323)	*P* value
**Pre-treatment**				0.070
Surgery	93	29	64	
TACE	175	75	100	
Ablation	238	79	159	
**Gender**				0.458
Male	420	151	269	
Female	86	32	54	
**Type of hepatitis**				0.455
HBV	331	139	192	
HCV	49	15	34	
HBV+HCV	9	4	5	
No	67	25	42	
**Differentiation**				0.248
High	249	99	150	
Middle	207	67	140	
Low	50	17	33	
**Child-Pugh**				0.153
A	448	158	290	
B	58	25	33	
**Cirrhosis**				0.159
Yes	420	145	275	
No	58	38	48	

*Note: TACE; transcatheter arterial chemoembolization;

**Table 3:** Comparison of clinical parameters of RHCC patients between the low and high NLR groups (T- test).

Group	Age (year)	Number of tumor (n)	Size of tumor (cm)	AFP (µg/L)	CHE (u/L)
NLR≥2.14	60.4±11.1	2.1±1.1	2.7±1.7	802.6±1854.4	5424.0±1609.4
NLR<2.14	58.5±10.9	2.0±1.1	2.6±1.2	389.5±1781.4	5857.2±1648.5
*P*	0.052	0.180	0.370	0.053	0.089

*Note: AFP: a-fetoprotein; CHE: serum cholinesterase.

## Discussion

Recent evidence suggests that the development of cancers including HCC is closely related with inflammation and immunity status, and the liver is an immune organ itself [17]. HCC most commonly occurs in patients with chronic liver disease, which induces chronic inflammation and impaired immunity. Neutrophils and lymphocytes are simple, effective and typical markers of inflammation and immunity and are easily evaluated in the clinic. Previous studies have shown that a higher NLR is correlated with adverse survival outcomes in patients with various solid tumors, which reflects relatively depleted lymphocytes and impaired host immune response to malignancy [5], [10]–[13]. This study is the first to describe the relationship between NLR and cancer recurrence in RHCC patients following thermal ablation.

We found that NLR is an independent predictor for re-recurrence in RHCC patients following thermal ablation using a multivariate Cox proportional hazard model. The result was consistent with prior studies [6], [18]–[19]. However, in all of the previous studies, the cutoff values of NLR were not the same. The cutoff value of NLR for predicating the prognosis and recurrence was different for each cancer after diverse treatment [20]–[22]. The present study is the first report stating a cutoff value of NLR for predicting re-recurrence in RHCC patients. Using ROC curve analysis, we found that the best NLR cutoff value of 2.14 had a relatively high sensitivity and specificity for preoperative prediction in RHCC patients with a high risk of tumor recurrence after thermal ablation. Therefore, we considered RHCC patients with an NLR <2.14 as one group and patients with an NLR ≥2.1 4 as another group. We then compared the 1- and 3-year recurrence rates of these two groups and observed a significant difference between the two groups. Furthermore, we compared the outcomes of these two groups, and we found a significant difference in RFS between these patients.

Although RHCC patients with high NLR have more cancer recurrences, the mechanisms through which NLR affects cancer recurrence are undefined. There are several possible hypotheses regarding the relationship between high NLR and cancer recurrence. First, chronic inflammation in HCC patients may be caused by HBV/HCV virus infection and the tumor itself. The high level of neutrophils in circulation is associated with the expression of granulocyte colony-stimulating factor in tumor tissue and macrophage colony-stimulating factor in tumor adjacent tissue [23]–[25]. Macrophages were the dominant type of myeloid cells and expressed CD163, IL-6, IL-10 and IL-17 [26]–[27]. Mano Y et al. reported that the high expression of CD163, IL-6, IL-10 and IL-17 was associated with high NLR by immunohistochemical analysis [20]. The neutrophils secreted vascular endothelial growth factor into the circulation, and this secretion was related with HCC recurrence, invasion and metastasis [28]. Further examination is necessary to determine a clear mechanism. Second, the human immune status mainly depends on lymphocytes, and these cells are considered to be a surrogate marker for patients’ level of immunosuppression and nutritional status. Lymphocytes are also a prognostic factor for survival and recurrence in several cancers [19]. It was reported that reduced lymphocyte infiltration reflects impaired anti-tumor function [4]. A high NLR is a relative ratio of neutrophils to lymphocytes and indicates an imbalance in the inflammatory cascade and immune response to malignant tumors. Thus, tumor recurrence and metastasis may occur more frequently in patients with a high NLR.

The results of this study are limited by the retrospective nature of the analysis and the relatively small number of patients enrolled. Additionally, the mechanism of the relationship between NLR and re-recurrence in RHCC patients was not examined. Therefore, additional multicenter prospective studies are needed to confirm and advance the findings demonstrated in this study.

In conclusion, the results of this study suggest that RHCC patients with high preoperative NLR have poorer RFS following thermal ablation. The best cutoff value for predicating re-recurrence is 2.14, and this value may improve the clinical management of RHCC patients after thermal ablation.
